# Hyperlipidemia in the Setting of Primary Biliary Cholangitis: A Case Report and Review of Management Strategies

**DOI:** 10.7759/cureus.31411

**Published:** 2022-11-12

**Authors:** Ange Ahoussougbemey Mele, Riaz Mahmood, Henry Ogbuagu, Jason Fombi

**Affiliations:** 1 Internal Medicine, Northeast Georgia Medical Center Gainesville, Gainesville, USA

**Keywords:** pcsk-9 inhibitors, statin safety, hyperlipidemia treatment, hyperlipidemia, primary biliary cholangitis

## Abstract

Primary biliary cholangitis (PBC) is a chronic and progressive cholestatic autoimmune liver disease. Its characteristic is the destruction of intrahepatic bile ducts with portal inflammation and scarring. In the setting of cholestasis, there is a reduction in bile acid production and, consequently, decreased intestinal absorption of cholesterol. The result is the endogenous synthesis of cholesterol in the liver and the secretion of very low-density lipoprotein. Mixed hyperlipidemia can be challenging to manage, and the association with increased cardiovascular events remains unclear.

## Introduction

Primary biliary cholangitis (PBC) is a condition that predisposes patients to increased endogenous production of cholesterol [1]. It is, therefore, important to closely monitor them and actively treat their dyslipidemia [2]. The risk of cardiovascular complications is low in the absence of metabolic syndrome. We suggest initiating moderate-intensity statins in the presence of diabetes or pre-existing cardiovascular diseases (CVDs) and closely monitoring PBC in patients with pre-existing CVDs, diabetes, or primary hypercholesterolemia [3]. In the following case report, we describe the pathogenesis of hypercholesterolemia in patients with PBC. Moreover, we propose effective management strategies to tackle this condition.

## Case presentation

**Figure 1:**
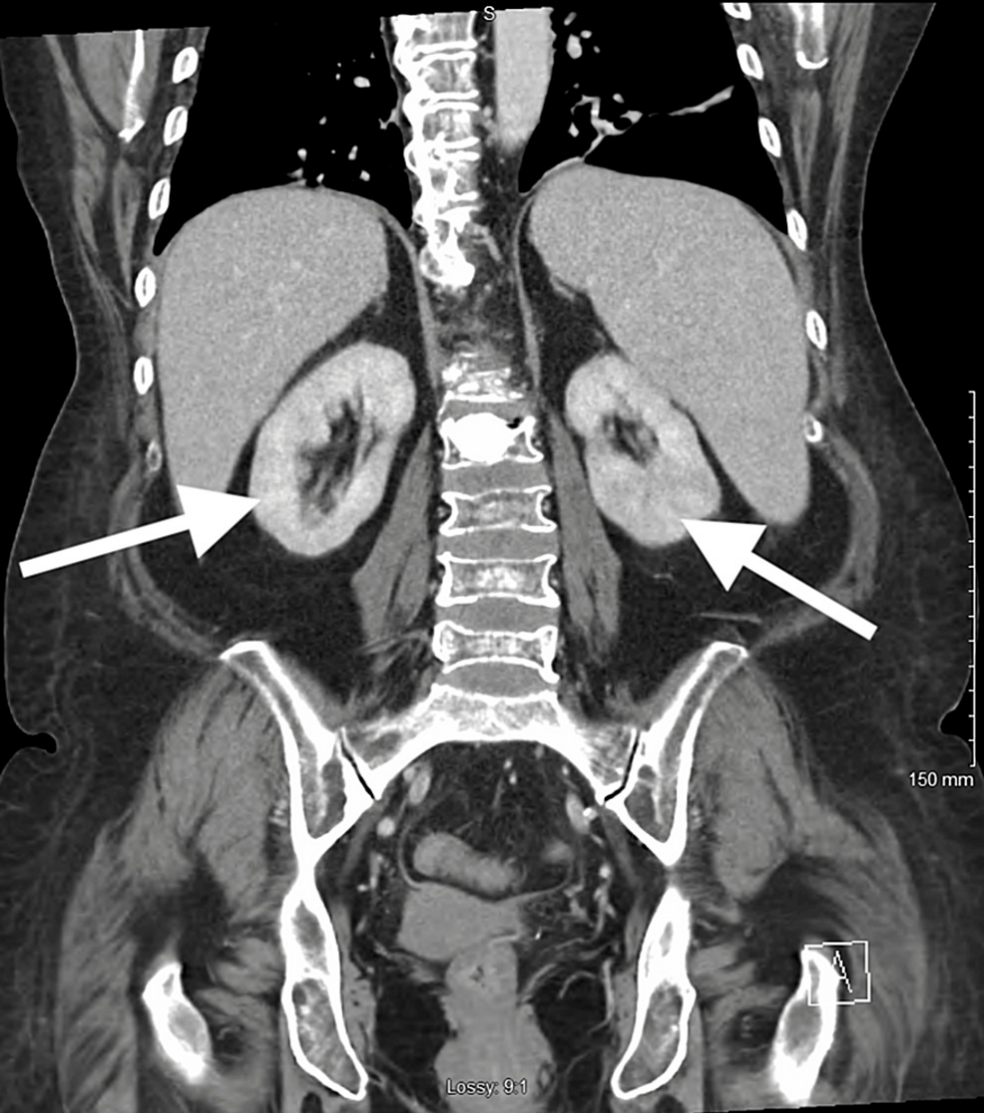
CT Abdomen and pelvis with contrast Findings consistent with evolving bilateral pyelonephritis as noted by white arrows. The left kidney demonstrates a striated appearance suggestive of evolving left pyelonephritis. The right kidney also demonstrates a striated appearance lesser than the left suggestive of evolving pyelonephritis.

We report a case of a 46-year-old female with a past medical history significant for recurrent urinary tract infections with multidrug-resistant organisms, diabetes mellitus type II, autoimmune hepatitis, systemic lupus erythematosus, and PBC. She presented to the Emergency Department with a chief complaint of dysuria. In the ED, vitals were significant for hypertension, with a blood pressure of 141/73 mmHg. Her physical examination revealed xanthoma of the eyelids and infra-umbilical abdominal pain. Lab workup revealed hemoglobin of 9.6 g/dL, white blood cells of 30.2 K/µL, aspartate aminotransferase 119 U/L, alanine aminotransferase 105 U/L, alkaline phosphate 825 U/L, total cholesterol of 689 mg/dL, triglycerides 301 mg/dL, low-density lipoprotein (LDL) of 342 mg/dL, and high-density lipoprotein (HDL) less than 20 mg/dL. Urine culture showed extended-spectrum beta-lactamase (ESBL) E. coli. Computed tomography abdomen and pelvis showed evolving bilateral pyelonephritis (Figure 1). She completed a 14-day course of ertapenem. She was discharged home with a primary care provider and Urology follow-ups.

The risk of cardiovascular complications is low in the absence of metabolic syndrome. We suggest initiating moderate-intensity statins in the presence of diabetes or pre-existing CVD and closely monitoring PBC in patients with pre-existing CVD, diabetes, or primary hypercholesterolemia.

## Discussion

PBC is a chronic and progressive cholestatic autoimmune liver disease. Its characteristic is the destruction of intrahepatic bile ducts with portal inflammation and scarring [1]. In the setting of cholestasis, there is a reduction in bile acid production and, consequently, decreased intestinal absorption of cholesterol. It results in the endogenous synthesis of cholesterol in the liver and the secretion of very LDL. Moreover, as described by Gylling et al., the composition of serum cholesterol in primary biliary cirrhosis is also altered by vesicles rich in phospholipid and unesterified cholesterol and low in cholesterol ester and triglycerides. These vesicles are called Lp-X. In the healthy liver, lecithin-cholesterol acyltransferase converts the free cholesterol in Lp-X into the cholesterol esters found in LDL. The reduced function of lecithin cholesterol acyltransferase in PBC results in changes in lipoprotein composition, decreased LDL, and increased Lp-X [1]. Lp-X protects endothelial cells and delays atherosclerosis by preventing LDL oxidation. Furthermore, Jahn et al. report that while early stages of PBC are associated with a mild elevation in VLDL and LDL and increased HDL, advanced stages of PBC are associated with elevated LDL with Lp-X and decreased HDL [2].

Longo et al. completed a cohort study of 400 PBC patients for 6.2 years which entailed serial determination of serum lipid levels and registration of all cardiovascular events. Disease progression was associated with a reduction in total (p<0.001) and HDL (p<0.05) cholesterol. In addition, the incidence of cardiovascular events was the same as that of the general population (cerebrovascular events: standardized ratio of 1.4, 95% confidence interval of (0.5-3.7); coronary events: (standardized ratio of 2.2; 95% confidence interval of 0.9-4.3). Hypertension results in an increased risk of cardiovascular events (standardized ratio of 3.8; 95% confidence interval of 1.6-8.9). Moderate hypercholesterolemia caused an increase in the risk of cardiovascular events (standardized ratio of 3.8; 95% confidence interval of 0.9-17). Severe hypercholesterolemia was not associated with increased risk (standardized ratio of 2.4; 95% confidence interval of 0.5-11) [3]. Floreani et al. assessed the influence of metabolic syndrome in response to ursodeoxycholic acid (UDCA) and survival in PBC patients. One hundred seventy-one PBC patients were eligible, and 55 had metabolic syndrome. Patients with metabolic syndrome had significantly more cardiovascular events (p<0.0001), and response to UDCA was more significant in the group without metabolic syndrome. The study concluded that when associated with metabolic syndrome, PBC should be monitored carefully due to the increased risk of cardiovascular events [4]. Consequently, hypercholesterolemia in PBC does not increase the risk of CVD unless there is concomitant metabolic syndrome [4].

Wah-Suarez et al. postulate that the initial assessment of patients with PBC should include screening for traditional risk factors such as tobacco use, diabetes, hypertension, elevated body mass index (BMI), and a lipid panel including ApoB-100 levels check [5]. The authors explain that conventional lipid panels do not distinguish Lp-X from LDL-cholesterol, making it impossible to use traditional LDL-cholesterol measurement for risk stratification. Because ApoB-100 is not present in Lp-X, it helps differentiate LDL-cholesterol from Lp-X. Reliance on LDL-Cholesterol from conventional lipid panels can overtreat hypercholesterolemia in the context of PBC. ApoB-100 is optimal for risk stratification [5].

Based on the Canadian Cardiovascular Society Guidelines, Wah-Suarez et al. recommend treating hypercholesterolemia in PBC patients with pre-existing CVD [5]. The same recommendation applies to patients with hypercholesterolemia with ApoB-100 level > 120 mg/dL. Patients with PBC without these risk factors but who are smokers, diabetic, or have hypertension or an elevated BMI should undergo treatment when ApoB-100 level >90 mg/dL. Stone et al. recommend treatment based on 10-year ASCVD risk stratification with a high-intensity statin with Atorvastatin 40-80 mg daily or Rosuvastatin 20-40 mg daily to decrease LDL cholesterol by > 50% or moderate-intensity statins to decrease LDL cholesterol by 30%-50% [6]. As there is no data on risk stratification for patients with PBC, the recommendations are to start with moderate-intensity statins and titrate as clinically indicated. After assessing compliance, if there is no reduction in ApoB-100 as expected while the patient is compliant, the recommended second line is proprotein convertase subtilisin Kexin 9 (PCSK9) inhibitors [5].

In patients without cardiovascular risk factors and ApoB-100 < 120 mg/dL, the next step is to recheck the lipid panel, including ApoB-100, every five years. Patients without cardiovascular risk factors and ApoB-100 > 129 mg/dL should start treatment with a moderate-intensity statin. Patients with pre-existing CVD and diabetes should undergo treatment with a moderate-intensity statin. Patients who have hypertension or are tobacco users and have ApoB-100 > 90 mg/dL should start treatment with a moderate-intensity statin. In patients with hypertension and tobacco use and ApoB-100 < 90 mg/dL, the next step is to recheck the lipid panel, including ApoB-100, every year. After moderate-intensity statin initiation, it is important to recheck the lipid panel with ApoB-100 in three months. If there is an expected decrease of 30%-50% in the ApoB-100 level, recheck the lipid panel periodically and titrate as needed. If there is less than a 30% decrease in ApoB-100 after three months, assess compliance. If the patient is compliant, up-titrate statin and recheck ApoB-100 in three months. If less than 30%, consider adding PCSK9 inhibitors. If the patient is non-compliant, address the reasons for non-compliance [5].

Several studies have evaluated the efficacy and safety of statins in primary biliary cirrhosis. The consensus is that they are safe for chronic liver disease and effective in treating hypercholesterolemia in PBC. Rajab et al. reviewed the use of statins in a large cohort of PBC patients at Tufts Medical Center over 10 years. Fifty-eight of their PBC patients were on statins, and five were on Ezetimibe for an average of 41 months (3 to 125 months). Alanine aminotransferases (ALT) were measured every three months. The study concluded that statins were well tolerated by these patients without complaints of muscle pain, weakness, or increased ALT levels. Upon initiation of statins, ALT levels were slightly elevated (41.7 +/- 25.1 U/L) but were normal the last time these patients were seen (39.0 +/- 21.0 U/L) (P < 0.303). Moreover, serum cholesterol levels were decreased by 30% from 262 +/- 45 mg/dL to 181 +/- 14 mg/dL (P < 0.01) [7]. Per Cash et al., patients with PBC were randomized to receive 20 mg simvastatin (n=11) or placebo (n=10) for 12 months. After the trial, serum cholesterol in the simvastatin group was significantly lower compared to the placebo group (4.91 mmol/L vs. 6.16 mmol/L, p = 0.01) [8]. Furthermore, Kim et al. performed a systematic review and meta-analysis showing that statin use is associated with a lower risk of hepatic decompensation and mortality and might reduce portal hypertension in chronic liver diseases [9].

Concerning PCSK9 inhibitors, Zhang et al. conducted a meta-analysis of 25 randomized control trials demonstrating that Evolocumab was not associated with liver enzyme abnormalities compared to a placebo [10]. UDCA is the first-line treatment for PBC. It lowers total cholesterol, LDL-cholesterol, and VLDL without affecting HDL cholesterol or total triglycerides [5]. Poupon et al. showed that UDCA is a safe medication for cholestatic liver disease with limited side effects [11].

## Conclusions

In conclusion, statins play a significant role in managing hypercholesterolemia in PBC. Several studies have demonstrated their safety and efficacy in decreasing levels of ApoB-100. PBC patients with metabolic syndrome and hypercholesterolemia require treatment and close follow-up. ApoB-100 level plays a vital role in managing hypercholesterolemia in PBC decision-making. A risk stratification system is needed to manage patients with PBC appropriately.
